# Supplementary material for: HTLV-1 Tax plugs and freezes UPF1 helicase leading to nonsense-mediated mRNA decay inhibition
Source: Nat Commun. 2018 Jan 30;9:431. doi: 10.1038/s41467-017-02793-6 (PMC5789848; doi:10.1038/s41467-017-02793-6)
Supplement: Supplementary file 1 — Supplementary Information [file 41467_2017_2793_MOESM1_ESM.pdf]

Supplementary Figure 1

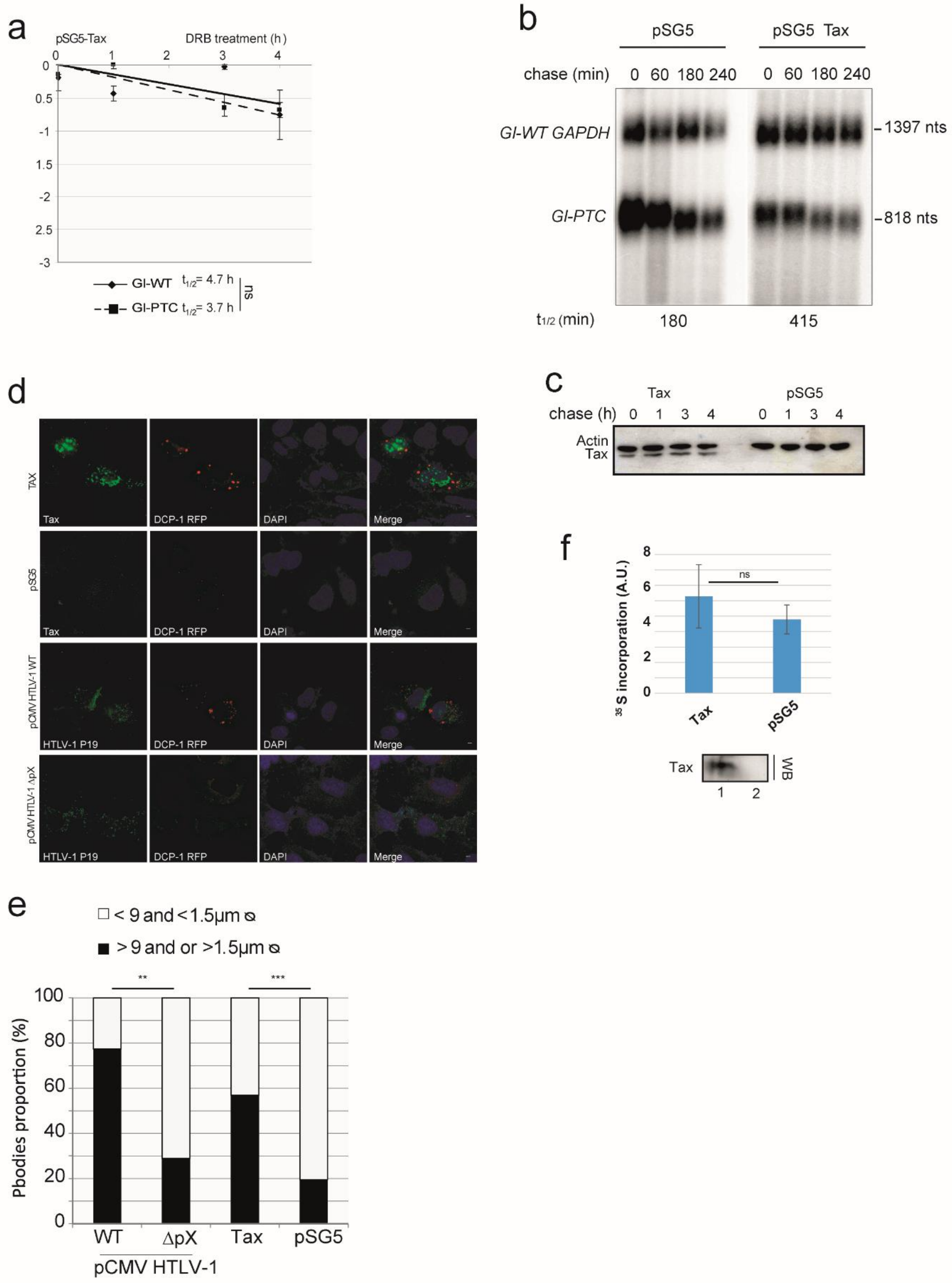

Supplementary Figure 1

Tax is directly involved in HTLV-1-mediated NMD inhibition. (**a**) RNA decay assays were carried out in HeLa cells. The stability of GI-PTC and GI-WT mRNA was analysed in the presence of Tax, after RNA quantification by qRT-PCR. mRNA half-lives ($t1/2=\ln(2)/\lambda$ with λ the time constant of the decay curves) are indicated in front of their respective conditions. Here we confirmed that Tax was able to stabilize the NMD sensitive GI-PTC mRNA to similar levels as the NMD insensitive GI-WTmRNA. (**b**) Northern blots showing the decay of GI-PTC mRNA in HeLa tet-off cells transfected with control and Tax expressing vectors. Numbers above the panel refer to hours after tetracycline-mediated transcriptional shut-off of GI-PTC mRNA (chase). The half-lives (*t*1/2) of globin mRNAs were calculated after normalization of levels of GI-PTC mRNA to levels of GI-WT–GAPDH fusion mRNA (GI-WT-GAPDH). The RNA lengths are indicated on the right (nucleotides, nts) excluding polyA-tails. (**c**) Western blot analysis of Tax expression during the RNA decay assay. (**d**) Confocal microscopy analysis of P-bodies formation in HeLa cells expressing DCP1-RFP(Red fluorescence) with a x63 objectif. Cells were also cotransfected with Tax, pSG5 (empty vector), the pCMVHTLV-1 WT and pCMVHTLV-1 ΔpX molecular clones as indicated on the left. Tax and p19 were visualized in green as indicated on the lower left angle of each view. Nucleic acids were also stained with DAPI (4′,6-diamidino-2-phenylindole; blue fluorescence). (**e**) Quantification revealed the percentage of aberrant P-bodies (number of P-bodies> 9 or P-body diameter >1.5 µm), compared to control profile. (**f**) Metabolic labelling using ^{35}S methionine was carried out in HeLa cells. ^{35}S labelled proteins levels were quantified and normalized according to coomassie staining levels. The expression of Tax does not significantly modify the levels of protein neosysnthesis. (**g**) Western blot controlling the expression of Tax in the metabolic labelling experiment.

The values represented in each graph correspond to the mean of at least three biological replicates, and the error bars correspond to the standard deviation. ns:P>0.05; ** P<0.01; ***P<0.005 with Student’s t test (two-tailed, unpaired).

Supplementary Figure 2

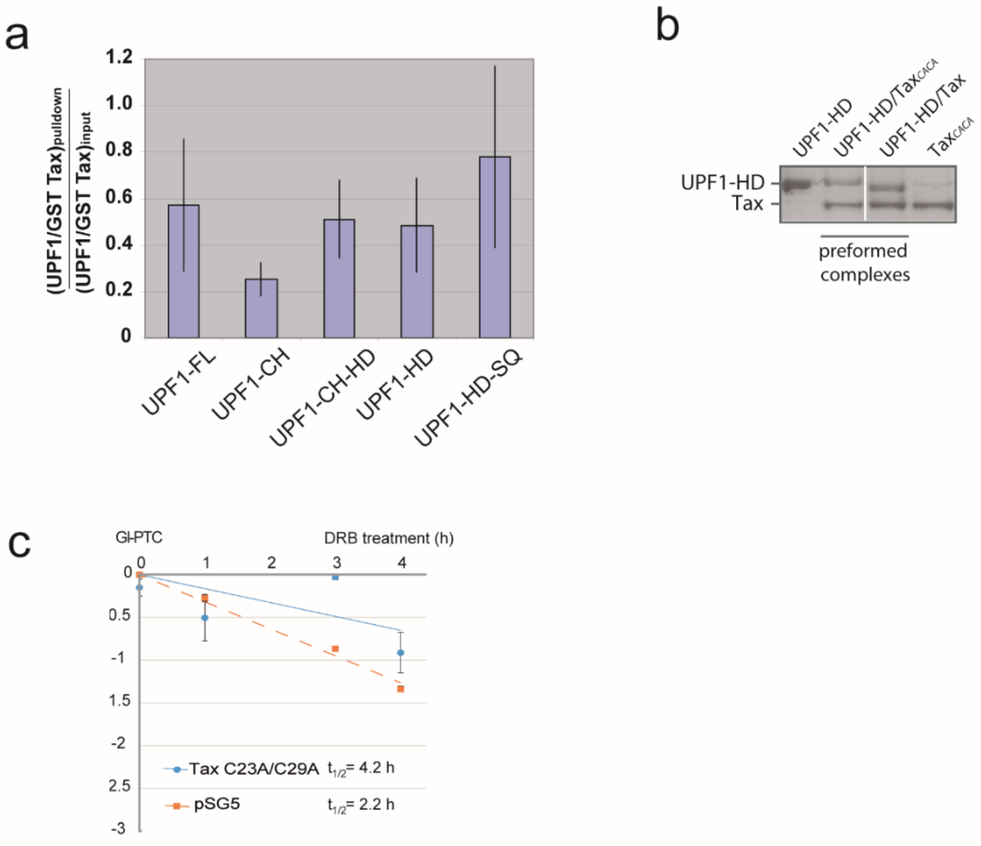

Supplementary Figure 2

Tax interacts directly with UPF1. (**a**) Quantification of GST pulldown assay showed in Fig. 2b. The fractions of UPF1 co-precipitated were normalized against the input. (**b**) Coomassie blue staining of proteins and protein complexes used for ATPase assay showed in Fig. 2d. (**c**) RNA decay assays of GI-PTC mRNA in HeLa cells expressing Tax_{CACA} (blue circle), or not (orange square).

The values represented in each graph correspond to the mean of at least three biological replicates, and the error bars correspond to the standard deviation.

Supplementary Figure 3

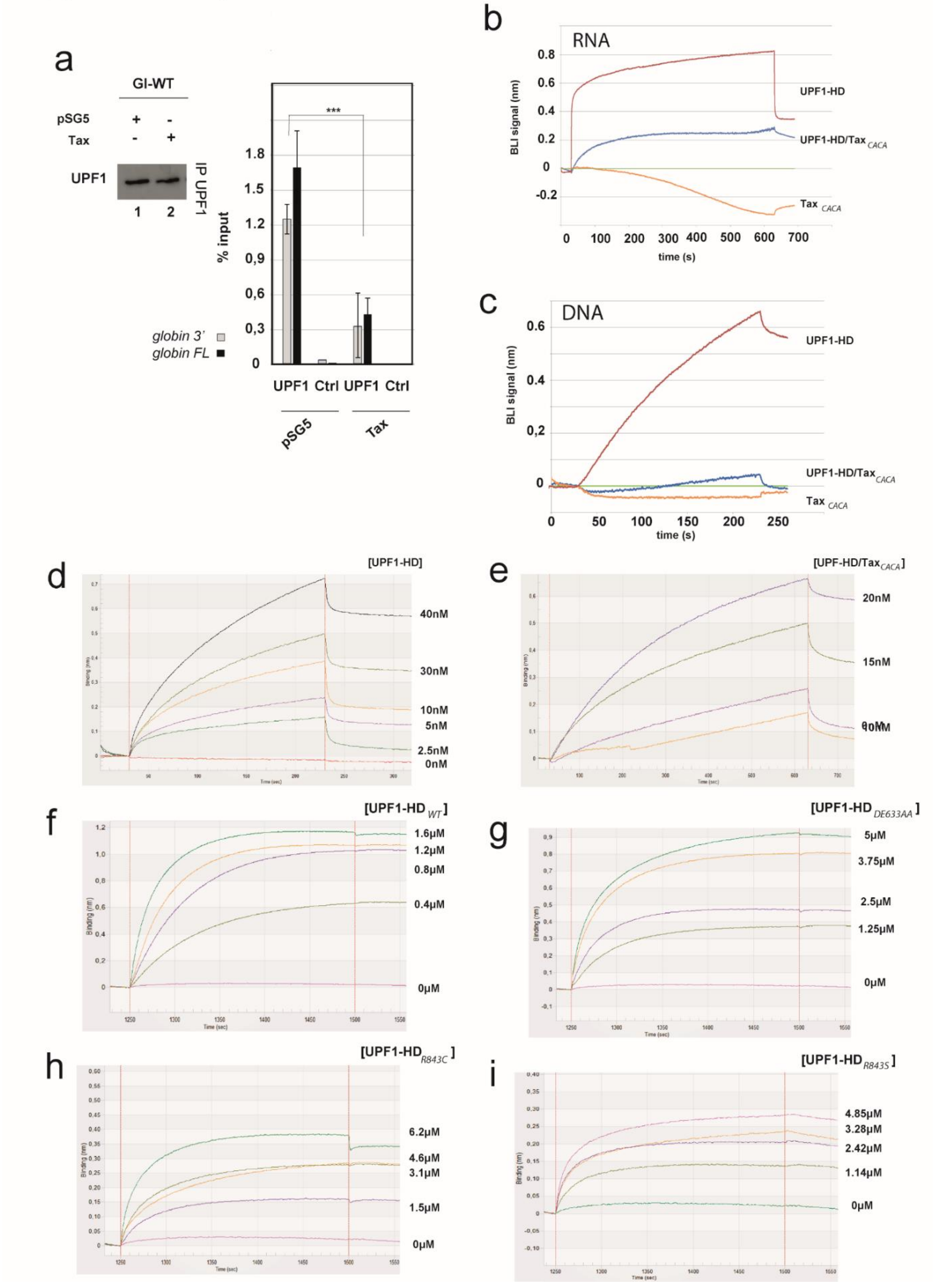

Suppplementary Figure 3

UPF1-HD/Tax complex possesses a lower affinity for RNA than UPF1-HD. (**a**) In the left panel, endogenous UPF1 immunoprecipitation (IP) from mock (lane 1) and Tax-expression vector (lane 2) transfected Hela cells. The level of immunoprecipitated UPF1 was analysed by western blot using anti-UPF1 antibodies. In the right panel, the histogram represents the quantification of relative GI-WT RNA recovery upon UPF1 IP (RNA Immuno Precipitation, RIP). The ΔΔCt method of mRNA quantification was applied to each experimental condition and oligonucleotide pairs used for RT amplification. The control sample (Ctrl) derived from IP using Immunoglobulin g (IgG) antibody. The values represented in each graph correspond to the mean of at least three biological replicates, and the error bars correspond to the standard deviation ***P<0.001 with a Student's t test (two tailed, unpaired) (**b**) Real-time sensorgram of Bio-Layer interferometry experiment showing the UPF1-HD binding to a 3'-end biotinylated 30 mer-RNA (left panel) and (**c**) 5'-end biotinylated 30 mer-DNA (right panel). The proteins or protein complexes used are indicated on the right of each curve. (**d**) Sensorgram of different concentrations of UPF1-HD and (**e**) UPF1-HD/Tax$_{CACA}$. (**f**) Sensorgram of different concentrations of UPF1-HD$_{WT}$, (**g**) UPF1-HD$_{DE633AA}$ (**h**) UPF1-HD$_{R843C}$ *and* (**i**) UPF1-HD$_{R843S}$. Those data were used to extract KD, *Kon* and *Koff* values, presented in Fig. 3.

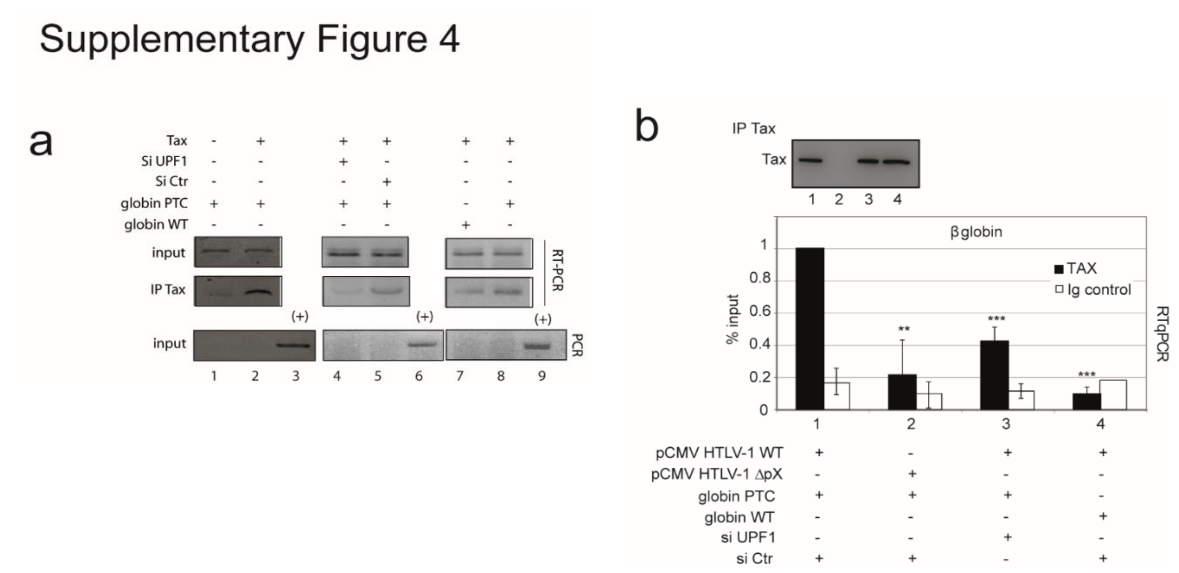

Supplementary Figure 4

Tax expressed from a HTLV-1 molecular clone precipitates mRNA substrates in a UPF1-dependent manner. (**a**) semi quantitative RT-PCR (24 cycles) on the RNA samples from RIP Tax experiment of Fig. 4a (upper panels). Amplified cDNA from input and IP Tax fractions were separated on a 1% agarose gel as indicated. DNA contaminations were checked by carrying out a PCR reaction without RT on the input sample. A positive control for PCR was added on lanes 3, 6 and 9 (lower panels) (**b**) Quantification of precipitated βglobin RNA from Tax immunoprecipitation (black bars) versus IgG immunoprecipitation control (white bars) from HeLa cells expressing pCMVHTLV-1 WT or pCMVHTLV-1 ΔpX. The histogram represents the quantification of relative RNA recovery upon normalization to input RNA of condition 1 that was settled to 1. The ΔΔCt method of mRNA quantification was applied to each experimental condition. The values represented in each graph correspond to the mean of at least three biological replicates, and the error bars correspond to the standard deviation; ** P<0.01; ***P<0.005 with Student's t test (two-tailed, unpaired).The western blot shows the amount of Tax immunoprecipitated in the condition used for RIP experiment described above.

Supplementary Figure 5

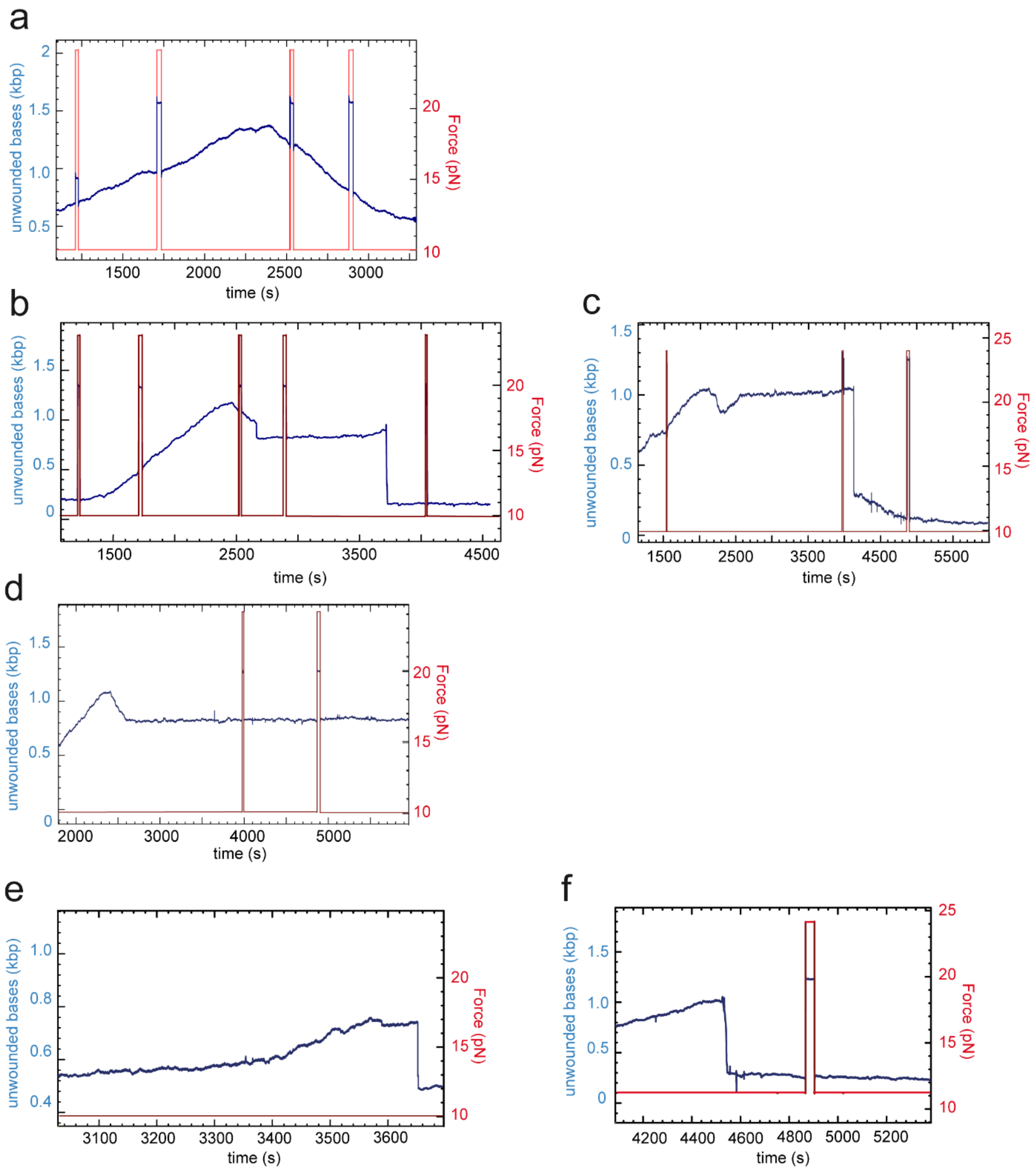

Supplementary Figure 5

Magnetic Tweezers recordings. This figure presents additional MT traks monitoring UPF1-HD activity in presence of Tax. **(a)** Trace of UPF1-HD activity. **(b-c)** Block of UPF1-HD translocation (between 2700s and 3700s in b pannel and between 2700s and 4200s in c panel) followed by substrate dissociation. **(d)** Block of UPF1-HD translocation at 2700s up to the end of this record track. This exemple shows that UPF1-HD does not systematically dissociate from its substrate after Tax inhibition. **(e-g)** UPF1-HD unwinds the DNA hairpin before dissociation induced by Tax binding.

Supplementary Figure 6

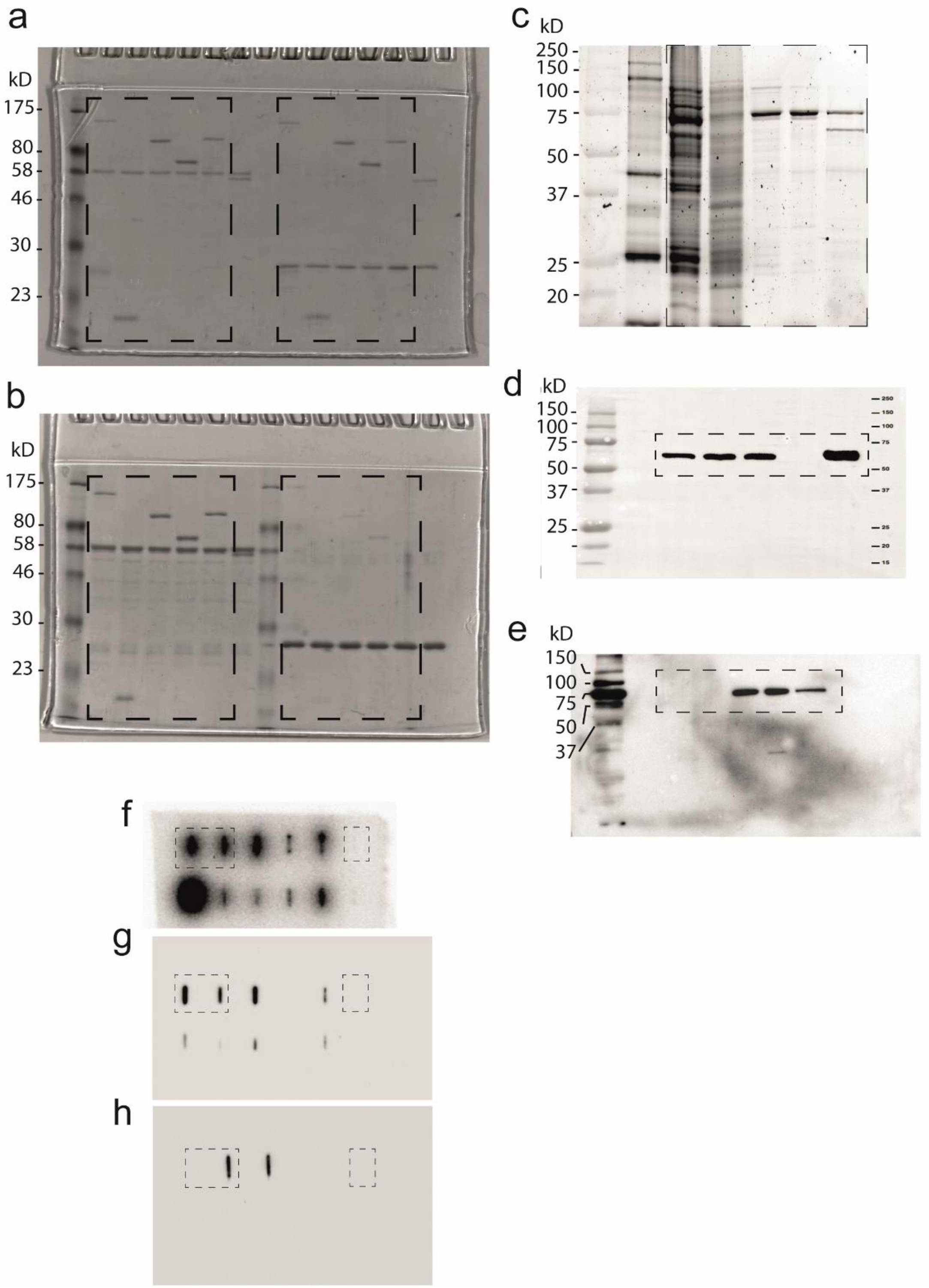

Supplementary Figure 6

Uncropped pictures related to figure 2. (a-b) uncropped gel from Fig 2b. **(c-e)** uncropped gel from Fig 2c. **(f-h)** uncropped gel from Fig 2e.

Supplementary Figure 7

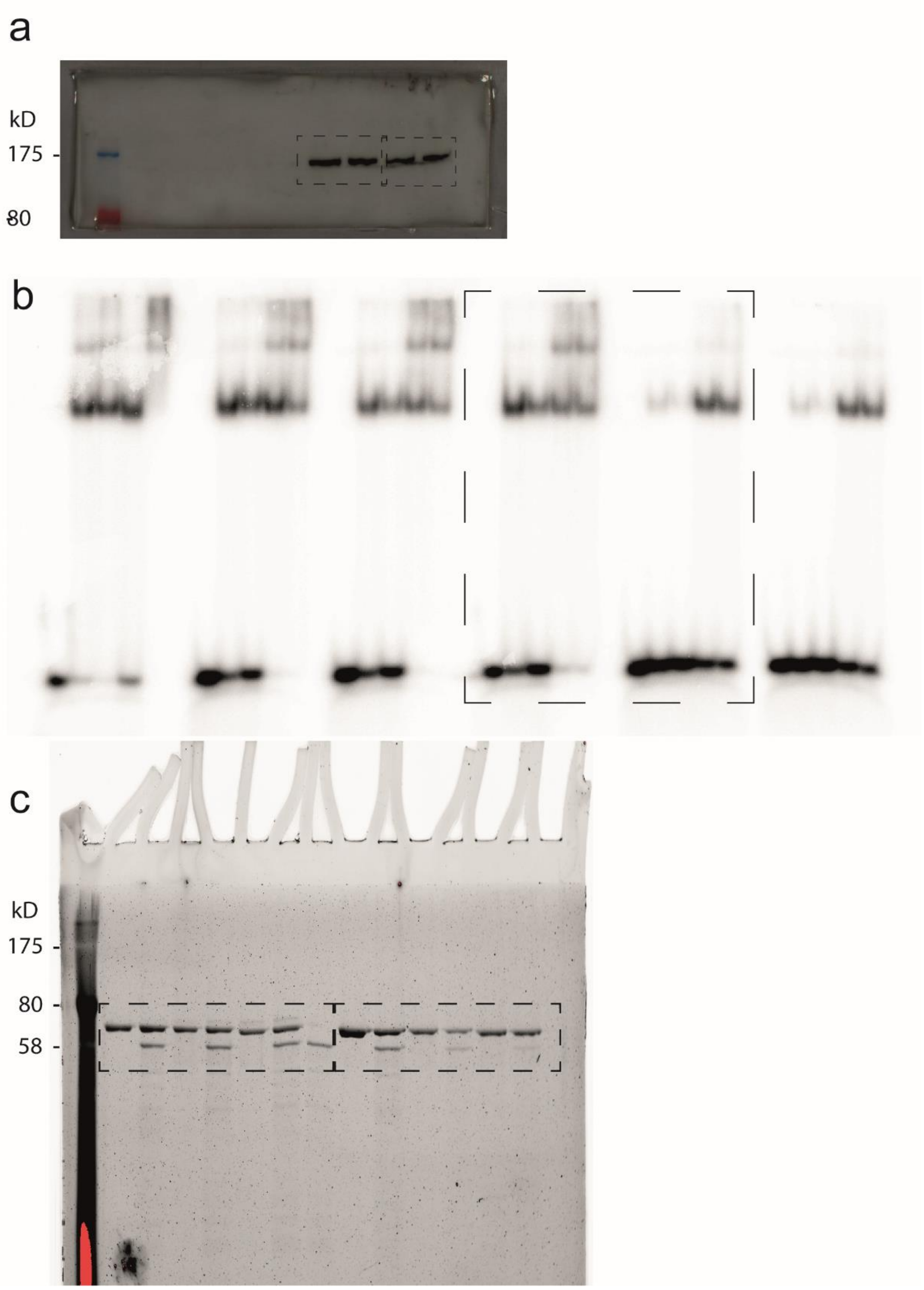

Supplementary Figure 7

Uncropped pictures related to figure 3. (a) uncropped gel from Fig 3a. **(b)** uncropped gel from Fig 3b. **(c)** uncropped gel from Fig 3e.

Supplementary Figure 8

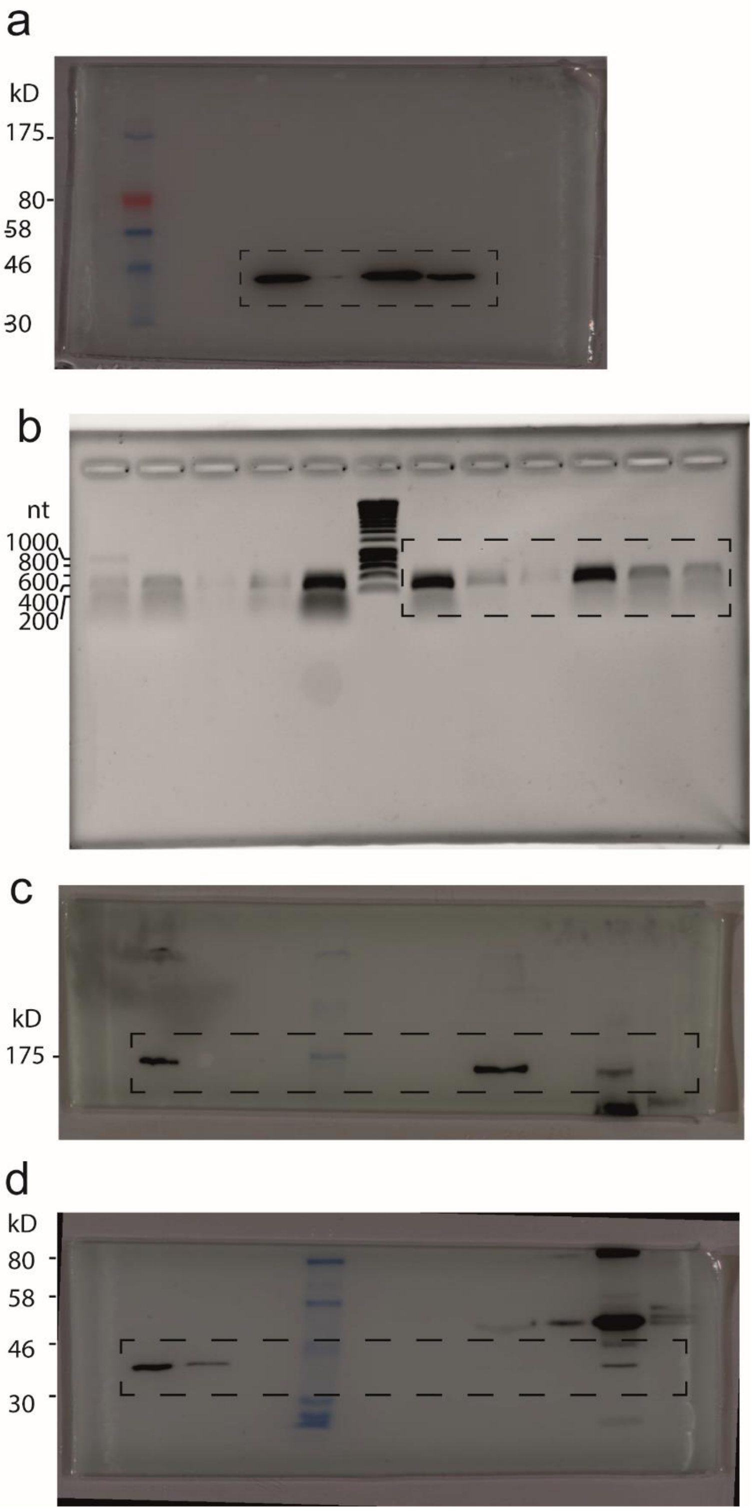

Supplementary Figure 8

Uncropped pictures related to figure 4. (a) uncropped gel from Fig 4a. **(b-c)** uncropped gel from Fig 4c. **(d)** uncropped gel from Fig 4d.

Supplementary Methods

Metabolic labelling

At 72h after transient transfection of control or Tax expression plasmids, HeLa cells were washed 3 times in 1x PBS and were further cultured in DMEM lacking methionine for 60 min. The cells were then incubated in DMEM supplemented with 0.1 $mCi.ml^{-1}$ of ^{35}S-labelled methionine and cysteine for 20 min. Whole cell extracts were prepared and analysed by SDS-PAGE on 10% polyacrylamide gels that were first stained with coomassie blue, and further analysed for radioactivity using a Typhoon phosphor imager system (GE Healthcare).

Magnetic tweezer

Sequence of the magnetic tweezer substrate:

5’ Biotin –
AATTGCATGTATTACTTGGTAGGATCCGTCATAGCTTTAGCGATTTGGGACACTTCATCAAGACTTCCAGAGCA
GCCGGAGACATATAGCTACAGGGGCCGCACTAGTGATTGCGGGATCCACATTATTACAAAATAAAGGGCTTCG
GCCCTTTAGCTTTATACGGAGTTTGATATAATGATATTTCTTGGATATGTGATACTTTTTCTTGCATTTTATCTAT
TCACTAGAGCATGTTGGATTGGGTTCTTTAGCACTCCAGATGGATTTATTTCAATAATTTTATTTTGCATTTCAAT
GACGGTTCTTGATATATGAAAATTTTAAATTTAGGTGATTGGCACTTAGGCGTTAAAGCTGATGATGAGTGGAT
TCGCGGTATTCAAATTGATGGAATTAAGCAAGCGATAGAATATTCTAAGAAAAATGGAATTACTACCTGGATTC
AATACGGTGATATTTTTGATGTGCGAAAAGCGATCACACATAATTCTATGGAGTTCGCCCGTGAAATAGTTCAT
TCGCTTGATGATGCTGGAATTACATTACACACTATTGTAGGAAATCACGATCTCCACTATAAAAATGTAATGCA
TCCAAATGCTTCTACTGAGCTTTTGGCTAAATATCCTAATGTTAAAGTGTATGATAAGCCTACTACAGTAGATTT
TGACGGGTGTTTGATTGATTTGATTCCTTGGATGTGCGAAGAAAATACTGGTGAAATTCTTGAGCATATCAGGC
GAGAGTTCTTTAGCCAGAAAGATTTCACAGAATTCCCAGTCATGAAGGTTACTATTGAAAATAATGATCAGGC
GTGGTCTTTATATCAGATGTTGAAAGCTTACTTTAAGGAATAATTATGCCGCTTTATGATTATAAATGTCAATCC
AAAGACTGTGCAAAAGAATACGAAAAAATCAAGAAAATTTCTGAAAGAGATACTGATGTATGTCCTGATTGTC
ATCGGCTGGCTGTTCGGTTAGTTTCCGCTCCTAAGCATGTGAATGGTGGATTTTACGACTTGCTTAAAGGGTAA
TTATGAAATATATTAATCGTTCTATTGCAGCATTAGTATTAGCAGTGTCTTTAATAGGATGTACTGATGCTGATA
ATGCTTCCAAAGTTTTGTCTTCAAGTGGTTTTACTAATATTGAAATCACTGGATATAATTGGTTCGGTTGCTCTG
AAAATGATTCCCAGCATACTGGATTTCGTGCTATTGGACCTACCGGGCAGAAAGTAGACCTCAGCGGATCCGC
GAATCCCGCGGCCATGGCGGCCGGGAGCATGCGACGTCGGGCCGTCAGATGCCTTTTGGCATCTGACGGCCC
GACGTCGCATGCTCCCGGCCGCCATGGCCGCGGGATTCGCGGATCCGCTGAGGTCTACTTTCTGCCCGGTAGG
TCCAATAGCACGAAATCCAGTATGCTGGGAATCATTTTCAGAGCAACCGAACCAATTATATCCAGTGATTTCAA
TATTAGTAAAACCACTTGAAGACAAAACTTTGGAAGCATTATCAGCATCAGTACATCCTATTAAAGACACTGCT
AATACTAATGCTGCAATAGAACGATTAATATATTTCATAATTACCCTTTAAGCAAGTCGTAAAATCCACCATTCA
CATGCTTAGGAGCGGAAACTAACCGAACAGCCAGCCGATGACAATCAGGACATACATCAGTATCTCTTTCAGA
AATTTTCTTGATTTTTTCGTATTCTTTTGCACAGTCTTTGGATTGACATTTATAATCATAAAGCGGCATAATTATT
CCTTAAAGTAAGCTTTCAACATCTGATATAAAGACCACGCCTGATCATTATTTTCAATAGTAACCTTCATGACTG
GGAATTCTGTGAAATCTTTCTGGCTAAAGAACTCTCGCCTGATATGCTCAAGAATTTCACCAGTATTTTCTTCGC
ACATCCAAGGAATCAAATCAATCAAACACCCGTCAAAATCTACTGTAGTAGGCTTATCATACACTTTAACATTA
GGATATTTAGCCAAAAGCTCAGTAGAAGCATTTGGATGCATTACATTTTTATAGTGGAGATCGTGATTTCCTAC
AATAGTGTGTAATGTAATTCCAGCATCATCAAGCGAATGAACTATTTCACGGGCGAACTCCATAGAATTATGTG
TGATCGCTTTTCGCACATCAAAAATATCACCGTATTGAATCCAGGTAGTAATTCCATTTTTCTTAGAATATTCTAT
CGCTTGCTTAATTCCATCAATTTGAATACCGCGAATCCACTCATCATCAGCTTTAACGCCTAAGTGCCAATCACC
TAAATTTAAAATTTTCATATATCAAGAACCGTCATTGAAATGCAAAATAAAATTATTGAAATAAATCCATCTGGA
GTGCTAAAGAACCCAATCCAACATGCTCTAGTGAATAGATAAAATGCAAGAAAAAGTATCACATATCCAAGAA
ATATCATTATATCAAACTCCGTATAAAGCTAAAGGGCCGAAGCCCTTTATTTTGTAATAATGTGGATCCCGCAA
TCACTAGTGCggccCCTGTAGCTATATGTCTCCGCCCCCCCCCCTGTGTGTGTGTGTGGTTGTGTGGTGTGTGGT
TGTGTGTTGGTGGTTGCATACTTCCGGGAACGCAGCAAACAACACACAAACAACACCCAAACAACACCAAACA
ACACACAAACAACACCCAAACAACACCACACAACAC U(Dig)U(Dig)U(Dig)U(Dig)U(Dig)U(Dig)
